# Microelectromechanical Resonant Accelerometer Designed with a High Sensitivity

**DOI:** 10.3390/s151229803

**Published:** 2015-12-03

**Authors:** Jing Zhang, Yan Su, Qin Shi, An-Ping Qiu

**Affiliations:** School of Mechanical Engineering, Nanjing University of Science and Technology, Nanjing 210094, China; zhangjing3701@126.com (J.Z.); suyan@mail.njust.edu.cn (Y.S.); sqinhy@mail.njust.edu.cn (Q.S.)

**Keywords:** resonant accelerometer, SOI, micro-lever mechanism, sensitivity, MEMS

## Abstract

This paper describes the design and experimental evaluation of a silicon micro-machined resonant accelerometer (SMRA). This type of accelerometer works on the principle that a proof mass under acceleration applies force to two double-ended tuning fork (DETF) resonators, and the frequency output of two DETFs exhibits a differential shift. The dies of an SMRA are fabricated using silicon-on-insulator (SOI) processing and wafer-level vacuum packaging. This research aims to design a high-sensitivity SMRA because a high sensitivity allows for the acceleration signal to be easily demodulated by frequency counting techniques and decreases the noise level. This study applies the energy-consumed concept and the Nelder-Mead algorithm in the SMRA to address the design issues and further increase its sensitivity. Using this novel method, the sensitivity of the SMRA has been increased by 66.1%, which attributes to both the re-designed DETF and the reduced energy loss on the micro-lever. The results of both the closed-form and finite-element analyses are described and are in agreement with one another. A resonant frequency of approximately 22 kHz, a frequency sensitivity of over 250 Hz per g, a one-hour bias stability of 55 μg, a bias repeatability (1σ) of 48 μg and the bias-instability of 4.8 μg have been achieved.

## 1. Introduction

Microelectromechanical accelerometers can be found in numerous applications such as inertial navigation systems, gaming, smartphones and mobile devices [1]. These devices are very attractive for high-precision measurement applications due to their high sensitivity, frequency output and large dynamic range [2,3,4]. In a silicon micro-machined resonant accelerometer (SMRA), the acceleration is measured through the differential frequency shift originated by axial loading between the two pull and push double-ended tuning fork (DETF) resonators. This type of resonant accelerometer benefits from a direct frequency shift between the resonators when sensing the input acceleration and this feature draws the extensive attention of researchers.

The sensitivity of the SMRA is defined as the differential output frequency of the resonators produced by an acceleration of 1 g. It is an important characteristic and deserves to be researched. A high sensitivity allows for the acceleration signal to be easily demodulated by frequency counting techniques [2] and decreases the overall noise on the readout electronics [5]. Recently, the design and fabrication of various mechanical resonant accelerometers have been studied [1,6,7]. Pinto *et al.*, have presented the design of a very small and sensitive resonant accelerometer [4]. By using thin silicon-on-insulator (SOI)-based technologies compatible with “In-IC“ integration, the accelerometer size has been reduced drastically (0.05 mm^2^ × 4.2 μm) with a sensitivity of 22 Hz/g. Sandia National Laboratories have developed an in-plane microelectromechanical systems (MEMS)-based nano-g accelerometer with a subwavelength optical resonant sensor in [8], where the authors focus on the maximum mass and the minimum spring constant to achieve a high sensitivity of 590 V/g and resolution of 17 ng/√Hz. Zou *et al.*, optimized a tilt accelerometer to obtain a design trade-off between sensitivity, resolution and robustness [9,10]. However, each part of this sensitive structure was optimized separately without considering the interaction effect between each other. Su *et al.*, designed a two-stage micro-leverage mechanism in the silicon resonant accelerometer and provided the theory for the amplification factor of the micro-leverage [11]. Constraint conditions have great effect on the amplification efficiency of the micro levers, but this study did not take these factors into consideration.

Although many of these studies have been concerned with the structure of resonant accelerometers to improve the ability to sense accelerations, their methods for the design and the sensitivity achievable from such sensors still remain limited. There remains a need for an efficient and systematic method that can obtain a reasonable structure with a high sensitivity based on the trade-off between the geometry of the accelerometer and its fabrication requirements.

This paper will show an in-plane SMRA by building upon previous work [5,12,13,14]. However, the geometrical setting, and hence, the properties of the mechanical parts are different. The structure of an SMRA is regarded as an energy transmission system, and each part consumes and transmits energy. Our study applies the energy-consumed concept to the SMRA to address the design issues and to increase its sensitivity. This sensor is referred to as a compliant mechanism. Based on the law of the conservation of energy, micro-lever mechanisms with boundary conditions are optimized to consume low energy and to show high force transmission efficiency between the proof mass and the resonators. This is very important for the design of resonant accelerometers. Currently, such an application of the energy-consumed concept has not been reported. In addition, the Nelder-Mead method [15,16] with constraint conditions is initially used as the optimization algorithm. The SOI processing has an integrated 80 μm-thick single-crystal silicon structure with a standard on-chip circuit. It offers a higher aspect ratio MEMS structure that will reduce the cross-axis sensitivity and increase the robustness of the sensors [17].

This paper demonstrates an SMRA with a 211.5 Hz/g nominal sensitivity (66.1% higher than the previous structure), one-hour bias stability of 55 μg and a bias repeatability of 48 μg. The device possesses a good output frequency nonlinearity of within ±40 g input acceleration (corresponding over the 16 kHz output frequency shift of the resonators). A very good agreement is obtained between the results of the closed-form analyses and those of the finite-element analyses. In what follows, we report on the experimental characterization of an SMRA based on the resonant sensing principles [2].

This paper is organized as follows: Section 2 describes the operation principle and the SOI processing for the SMRA dies, Section 3 proposes the reasonable design for each part of the SMRA and how to obtain an optimum sensitivity, Section 4 presents simulated and experimental results to compare with the theoretical predictions, and Section 5 contains the conclusions of the entire work.

## 2. Background

### 2.1. Operation Principle

**Figure 1:**
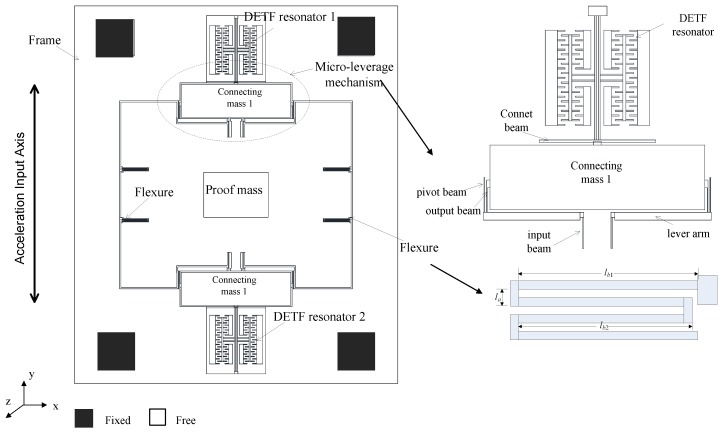
Schematic view of the SMRA with a frame structure.

The SMRA structure is shown as a schematic in Figure 1. This structure can be divided into four major components: a proof mass, micro levers, flexure suspensions and DETFs. All these components have coplanar faces. Two DETFs are joined via micro-levers to the proof mass. The proof mass is constrained to move along the y-axis by four flexures, which are linked to the frame mounted to the silicon substrate by four anchors. When acceleration along the input axis is applied to the device, the force from the proof mass is magnified by micro-lever and then transferred to the DETFs. This input applies axial loads, either tension or compression, to the DETFs, which produces a measurable natural frequency shift between the two resonators. The output of the SMRA is the differential frequency variation of the two resonators, and this differential arrangement enables a first-order cancellation of common parasitic sensitivities such as temperature.

### 2.2. Dies Based on the SOI-MEMS Process

The SMRA has been fabricated with SOI processing and wafer-level vacuum packaging [18]. The main characteristic of the SOI process is the use of silicon-to-silicon direct bonding (SSDB) and high-aspect ratio inductively coupled plasma (ICP) etching technology [17,19]. This process offers an 80 μm-thick MEMS structure with a high aspect ratio up to 1:30, which will then reduce cross-axis sensitivity and increase the robustness of the sensors.

**Figure 2:**
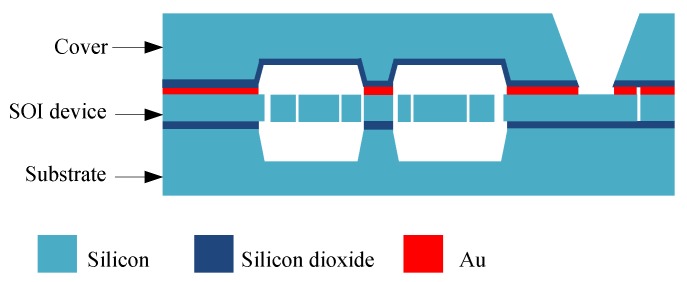
SOI-MEMS (silicon-on-insulator microelectromechanical system) process cross-section of the die.

**Figure 3:**
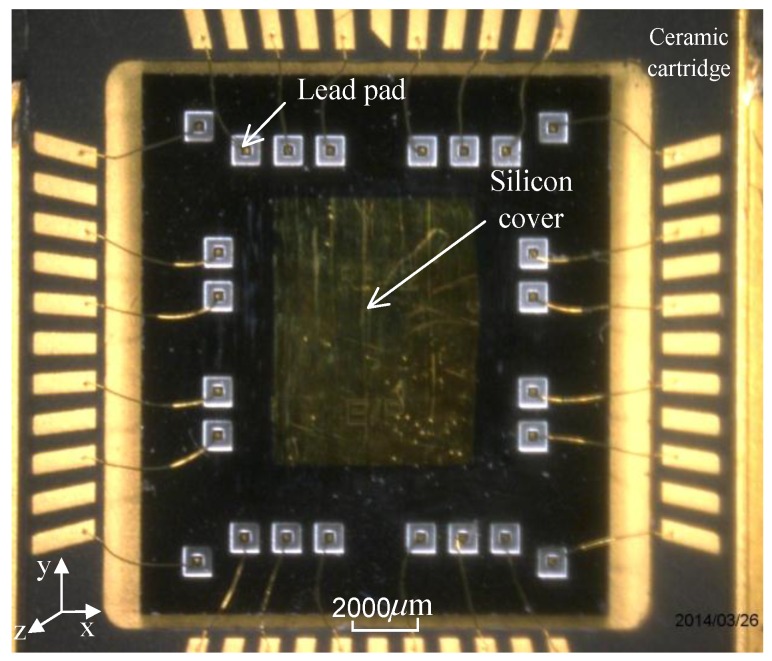
Photo of the SMRA wafer-level vacuum packaged die.

**Figure 4:**
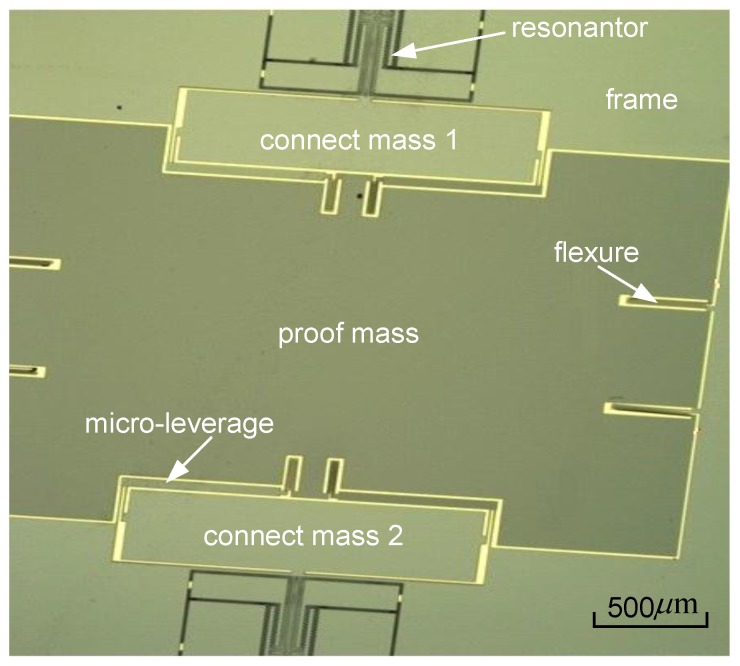
SEM photo of the mechanical sensitive structure.

The process cross-section is schematically represented in Figure 2. The die is realized with three wafers, the substrate, the SOI device layer and the cover. The sensor surface is around 110 mm^2^ and its thickness for the three layers is 700 μm. The SOI device layer is 80 μm thick and is manufactured using deep reactive ion etching on the SOI wafer with a high aspect ratio of up to 1:30. The residual stress is much less than that of a silicon-on-glass (SOG) process [20]. The cover and the active SOI layer are joined by an Au/Si eutectic bonding, forming a hermetic cavity that maintains the vacuum needed for a high-Q operation of the SMRA. To maintain the vacuum level over the long term, a getter is adhered to the inner surface of the cover, and once it is activated, the getter progressively absorbs and traps gaseous species. The packaged SMRA die was placed in a ceramic cartridge to protect the silicon structure and to facilitate the welding of the whole device. Figure 3 shows the wafer-level vacuum packaged SMRA dies, and Figure 4 shows the SEM (Scanning Electron Microscope) photo of the SMRA structure. The vacuum level of the sensor is measured at 10 Pa.

## 3. Device Design

### 3.1. Theoretical Analysis

For each DETF, the natural frequency of the basic lateral vibration mode is expressed as [21]

(1) $$f_{0}=\frac{1}{2\pi }\sqrt{\frac{16.55Et{\left(\frac{w}{l}\right)}^{3}}{0.397\rho wtl+\rho q_{s}t}}=\frac{1}{2\pi }\sqrt{\frac{16.55E{\left(\frac{w}{l}\right)}^{3}}{0.397\rho wl+\rho q_{s}}}$$

where *E* is the Young’s modulus, *ρ* is the density of single-crystal silicon and *l*, *w* and *t* are the length, width and thickness of the resonant beam, respectively. The comb-drive structure and the resonant beam have the same thickness, and *q*_s_ is the x-y plane area of the comb-drive structure. If *q*_s_ is constant, it is clear that the natural frequency of one resonant beam depends on the length and the width but is independent of the thickness of the beam.

When the acceleration *a* along the sensitive axis is applied (see Figure 1), the proof mass is subjected to the force *F*_1_ = *m*_1_*a*, and each of the connecting masses is subjected to *F_2_* = *m_2_a*. The axial force on each resonant beam has been magnified by the micro-lever to be

(2) $$F=-\left(\frac{A^{*}m_{1}a}{4}+\frac{m_{2}a}{2}\right)$$

where A^*^ is the amplification factor of the sensitive structure (*i.e.*, the effective amplification factor) and *m*_1_ and *m*_2_ are the mass of the proof mass and the connecting mass.

Therefore, the resonant beam frequency *f* under acceleration can be found by energy analysis [22] to be

(3) $$f=\frac{1}{2\pi }\sqrt{\frac{K_{eff}}{M_{eff}}}=\frac{1}{2\pi }\sqrt{\frac{16.55Et{\left(\frac{w}{l}\right)}^{3}\pm 4.85\frac{F}{l}}{0.397\rho wtl+m_{s}}}=f_{0}\sqrt{1\pm \frac{0.073\left(A^{*}m_{1}+2m_{2}\right)al^{2}}{Ew^{3}t}}$$

where *M_eff_* is the effective mass and *K_eff_* is the axial effective stiffness of the DETF.

Setting $\alpha =\frac{0.073\left(A^{*}m_{1}+2m_{2}\right)al^{2}}{Ew^{3}t}$, the frequency shift between two DETFs is

(4) $$\Delta f=f_{0}\sqrt{1+\alpha }-f_{0}\sqrt{1-\alpha }=f_{0}\alpha +\frac{1}{8}f_{0}\alpha^{3}$$

By substituting *a* = *n*g (where *n* is the applied acceleration in terms of *g*) into Equation (4) and by taking the derivative of *Δf* with respect to *n*, the sensitivity can be expressed in terms of frequency (with units of Hz/g)

(5) $$S_{g}=\frac{d\Delta f}{dn}\approx f_{0}\frac{0.073\left(A^{*}q_{1}+2q_{2}\right)\rho l^{2}}{Ew^{3}}\text{g}=0.0473\sqrt{\frac{l}{\left(0.397\rho wtl+m_{s}\right)Ew^{3}t}}\left(A^{*}m_{1}+2m_{2}\right)\text{g}=S_{res}\left(A^{*}m_{1}\text{g}+2m_{2}g\right)$$

where *S*_res_ is the sensitivity of the DETF sensing element.

(6) $$S_{res}=0.0473\sqrt{\frac{l}{\left(0.397\rho wtl+m_{s}\right)Ew^{3}t}}$$

### 3.2. Effective Amplification Factor A^*^

When acceleration *a* along the input axis is applied to the device, the force from the proof mass is magnified by the micro-lever and then transferred to the DETFs. The effective amplification factor A^*^ is therefore defined as the ratio of the axial force of the DETF beam to the input inertial force of the proof mass. Because the structure is symmetrical with respect to both the *x*- and *y*-axes, only one-quarter of the structure has been directly analyzed.

**Figure 5:**
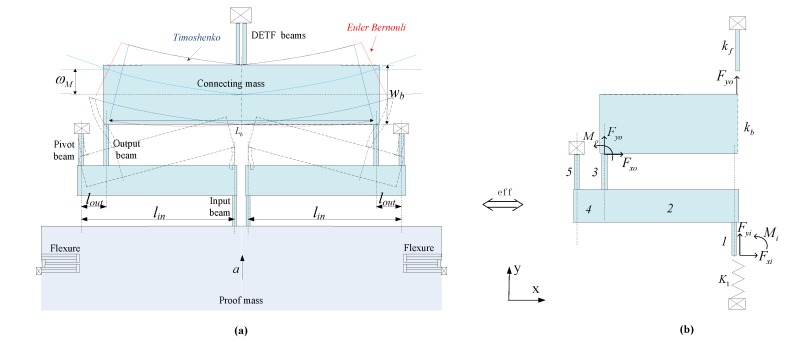
(**a**) Deformation of the micro-leverage mechanism; (**b**) The equivalent micro lever under loading

Figure 5a shows the model and deformation of each part of the half structure under an inertial load *a*. All the deformations have been exaggerated for clarity. Because the connecting mass is symmetrical with respect to the y-axis, the bending moment and the horizontal force transferred from output beams will be counteracted. As a result, only the axial force can be transferred to the DETFs and will therefore cause vertical displacements. Figure 5b shows the equivalent model of a quarter of the structure. By supposing the flexure can be regarded as a vertical spring *K*_1_, half of the connecting mass and one DETF can be regarded as vertical springs of which stiffness are *k_f_* and *k_b_*.

By solving the boundary conditions for *F*_xi_, *F_yi_*, *M_i_*, *F_yo_*, *F*_xo_ and *M_o_* (see Appendix A), the effective amplification factor can be obtained to be

(7) $$A^{*}=\frac{F_{yo}}{m_{1}a/4}$$

Because this is a fairly large output, the expression for A^*^ is shown in Appendix A. Based on the analysis above, the sensitive structure should be designed to produce a high effective amplification factor A^*^, which proportionally contributes to the sensitivity.

By substituting Equation (A8) into Equation (A12), we obtain

(8) $$A^{*}=\frac{F_{yo}}{F_{yi}+K_{1}d_{yi}}=\frac{F_{yo}/d_{yi}}{F_{yi}/d_{yi}+K_{1}}=\frac{A_{lever}K_{lever}}{K_{lever}+K_{1}}$$

where *A_lever_* = *F_yo_/F_yi_* represents the amplification factor of the micro-lever, and *K_lever_* = *F_yi_/d_yi_* represents the spring constant of the micro-lever. Both *A_lever_* and *K_lever_* are decided by the geometry of the micro-lever, and *K*_1_ has no influence on them; therefore, the effective amplification factor A^*^ increases as *K*_1_ decreases. As for the connecting mass, it should be rigid to ensure energy conservation in an ideal situation. In reality, if *k_b_* is 10 times greater than *k_f_*, the connecting mass can be regarded as rigid. The design for the connecting mass relies on this principle.

### 3.3. DETF Design Analysis

**Figure 6:**
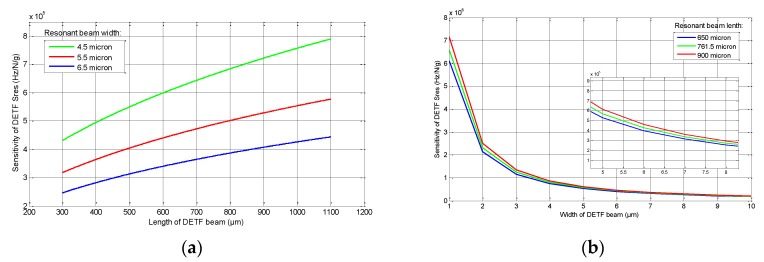
(**a**) *S*_res_ as a function of the DETF beam length for a series of beam widths; and (**b**) *S*_res_ as a function of the DETF beam width for a series of beam lengths.

The corresponding enhancement to the sensitivity owes not only to micro-lever mechanisms’ reasonable design, but also to the reasonable design for the resonators. As shown in Equation (6), the sensitivity of DETF *S*_res_ increases with an increase to the beam length and decreases with an increase to the beam width. Figure 6 shows the variation trend of *S*_res_: the sensitivity of DETF increases steadily when the beam length changes from 300 μm to 1100 μm, while it decreases rapidly when the beam width changes from 1 μm to 10 μm, especially when less than 3 μm. Thinner, longer beams provide more sensitivity, but may also cause resonator mismatch due to process, with detrimental effects to the temperature stability of the sensor. This is a trade-off in the mechanical structure design, and the optimum geometry of DETF will be point out in next section.

### 3.4. Reasonable Design of the Structure

**Figure 7:**
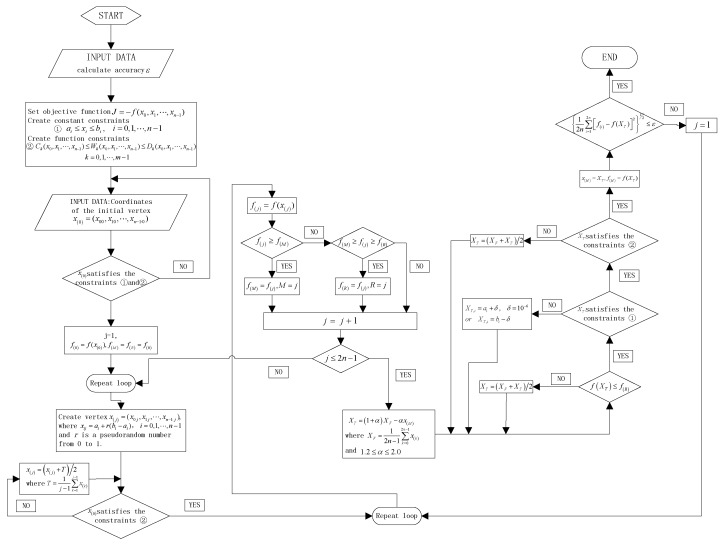
Flow chart for the Nelder-Mead method under constraint conditions.

By substituting Equation (7) into Equation (5), there is a tremendous dimension system about the sensitivity *S*_g_. To obtain a high *S*_g_, the Nelder-Mead method under constraint conditions is used as the optimization algorithm for each part of the SMRA. The Nelder-Mead method is a technique for minimizing an objective function in multidimensional space. It uses the concept of a simplex, which is a special polyhedron with N + 1~2N vertexes in N dimensions [16]. In this paper, the energy-consumed concept (energy-consumed concept: based on the conservation of energy law, micro-lever mechanisms with boundary conditions are optimized to consume a low amount of energy and show high-force transmission efficiency from the proof mass to the resonators) is applied to this method and used as the structure optimization algorithm. The flow chart for this algorithm under constraint conditions is shown in Figure 7. The sensitivity is regarded as the negative objective function to obtain a maximum result. Limited by the layout size, the proof mass area is assumed to be lower than 8 mm^2^. The first vibrating mode (the first mode: when SMRA is under acceleration, the proof mass will generate an inertial force, amplified by the micro-lever mechanism; then, the amplified inertial force will cause axial push and pull loading on the DETF resonators) frequency can be expressed as

(9) $$f_{1}=\sqrt{\frac{4\left(K_{lever}+K_{1}\right)}{m_{1}}}$$

In the testing environment, the first mode frequency should be larger than 2.1 kHz to stay away from low-frequency external vibrations. Substituting *m*_1_ into Equation (9), the spring constant *K*_1_ + *K_lever_* is assumed to be more than 71.4 N/m. The width of each beam is assumed to be equal to or larger than 4.5 μm which is limited by the processing level. Therefore, the range of each beam’s geometry is set as shown in Table 1 for our design requirements. Because *k_b_* is assumed to be 10 times greater than *k_f_*, the connecting mass can be regarded as rigid, which helps to reduce the energy consumed. All the above requirements are used as constraint conditions for the Nelder-Mead method.

The SMRA geometric dimensions after optimization are shown in Table 1. Its sensitivity is determined to be 216.35 Hz/g. Limiting by the layout size and processing level and based on the energy-consumed concept, several dimensions have been corrected slightly. The final sensitivity is 211.5 Hz/g, 66.1% higher than the previous structure’s sensitivity of 127.33 Hz/g [14]. The FEM (finite-element analyses) result of the first vibrating mode is 1994.59 Hz, 4.7% lower than the theoretical value of 2.1 kHz. Meanwhile, the energy consumed in each component of this sensitive structure is shown in Table 2. The DETF in this work consumes 59.6% of the total energy, while the DETF of the earlier structure consumes only 6.58 ppm (parts per million). Table 3 shows the ratio of the sensitivity improvement contributed by the DETF, the new micro-lever and the proof mass. This means that the sensitivity improvement is mainly attributed to both the re-designed DETF and the reduced energy loss on the lever. The micro-lever mechanisms (given the boundary conditions) consume lower energy and show high force transmission efficiency from the proof mass to the DETF resonators. If another optimum sensitivity is required, users can simply change the constraint conditions of the Nelder-Mead algorithm and repeat the steps above.

**Table 1:** Sensitive Structure Dimensions of the SMRA compared to earlier structure.

Variable	*l_r_* (μm)	*w_r_* (μm)	*l_i_* (μm)	*w_i_* (μm)	*l_o_* (μm)	*w_o_* (μm)	*l_p_* (μm)	*w_p_* (μm)	*l_in_* (μm)	*l_out_* (μm)	*w_c_* (μm)	*L_c_* (μm)	*l_a_* (μm)	*l_b1_* (μm)	*l_b2_* (μm)
Size range	100–1500	5.5–8	50–480	4.5–10	20–350	4.5–10	10–350	4.5–10	200–1650	>(*w_o_*+ *w_p_*)/2	*l_in_*/10 < *w_c_* < *l_in_*/2	2*l_in_*-10	9–20	200–1000	*L_b1_*–20
Optimal value	761.4	5.5	242.2	5.63	44.8	5.24	196.1	4.5	1641	54.9	1225	3272	10.7	390.3	370.3
Corrected value	761.5	5.5	242	5.5	45	5	196	4.5	900	29	525	1700	11	390	370
Earlier structure	1000	8	300	6	60	4	270	6	644	19	--	--	16	650	650

* *l* and *w*, respectively, represent length and width. *r*, *i*, *o* and *p* represent the length of the resonant beam, input beam, output system and pivot beam, respectively.

**Table 2:** Energy consumed in each component of the SMRA.

Component	Energy Consume × 10^−17^*a*^2^ (*J*)		Ratio (%)	
	Earlier Structure	This Work	Earlier Structure	This work
DETF	7.96 × 10^−4^	24.56	6.58 × 10^−4^	59.6
Flexure	41.93	6.51	34.64	15.8
Input beam	51.69	2.19	42.71	5.3
Output beam	23.35	1.09	19.3	2.6
Pivot beam	3.825	1.04	3.16	2.5
Lever arm(in)	0.24	2.55	0.198	6.2
Lever arm(out)	9.47 × 10^−5^	0.07	7.82 × 10^−7^	0.2
Connecting mass	8.8 × 10^−4^	3.27	7.27 × 10^−6^	7.9

**Table 3:** The sensitivity improvement between the two work.

SMRA	Sensitivity of DETF (Hz/N)	Effective Amplification Factor A^*^	The Proof Mass (kg)	*S*_g_ (Hz/g)
Previous design	399,393	22.04	1.42 × 10^−6^	127.33
This work	490,303	26.67	1.7 × 10^−6^	211.5
Ratio for the improvement	22.8%	21%	19.72%	66%

## 4. Simulation and Experiments for the SMRA

The micro-lever mechanism can be evaluated by the output frequency by supposing one resonator’s natural frequency is *f*_10_. When it is subjected to an inertial force, the frequency becomes *f*_1_. By substituting *f*_10_ into Equation (1) and *f*_1_ into Equation (3), we obtain

(10) $$f_{1}^{2}-f_{10}^{2}=\frac{0.073f_{10}^{2}\left(A^{*}q_{1}+2q_{2}\right)\rho al^{2}}{Ew^{3}}$$

By setting $c=\frac{0.073\rho al^{2}}{Ew^{3}}$, the effective amplification factor A^*^ for one resonator can be determine by Equation (10) as follows:

(11) $$A^{*}=\frac{f_{1}^{2}/f_{10}^{2}-\left(1+2q_{2}c\right)}{q_{1}c}$$

Equation (11) can be used to judge whether the design of the micro-lever mechanism is reasonable.

**Figure 8:**
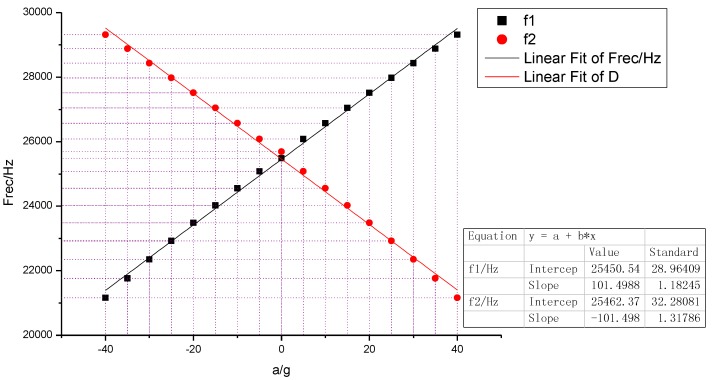
Simulated resonant frequency output *versus* the input acceleration.

The SMRA has been simulated by FEA software, from which the sensitive structure was subjected to an input acceleration in the range of ±40 g (see Figure 8). By fitting 13 datasets, the output frequency, simulated frequency and sensitivity agree with the designed values, as shown in Table 4. Moreover, the effective amplification factor is calculated through Equation (11). The nonlinearity of *S*_g_ within ±40 g is 49.66 ppm (parts per million).

**Table 4:** Simulated and testing results of the SMRA.

Results and Errors	*f_0_* (Hz)	Effective Amplification Factor A^*^	*Sg* (Hz/g)
Theory results	26,053.5	26.2	211.5
Simulated values	25,585.4	26.67/25.33	203
Relative shift	1.8%	1.88%/3.33%	4.19%

The initial testing was performed in open air at the Sci & Tech Micro Inertial Technology Lab of the Nanjing University. Three SMRA prototypes (A1-5, A1-7, and A1-8) had been chosen for testing these prototypes adopted a self-excited oscillation loop with automatic gain control (AGC) as the drive circuit, and the packaged SMRA dies were finally placed in a ceramic cartridge. The ceramic cartridge package was put on a socket that was wire-connected to an off-chip circuit on a PC board. During our testing, the output was connected to an oscilloscope. Without any input acceleration in A1-5, the resonant frequency of one DETF was 22,447.45 Hz and that of the other DETF was 22,179.4 Hz. The gaps between the normalized frequencies are attributed to thermal and residual stress during the process. Substituting the measured frequency into Equation (5), the theoretical sensitivity is determined to be 249.46 Hz/g.

The PC board of A1-5 was then placed vertically on a rotating platform with a constant temperature control. When this prototype was subjected to 1 g, the resonant frequency for the pull resonator was 22,574.23 Hz, while the push resonator was 22,047.41 Hz. The increased frequency of the pull resonator was 126.78 Hz, and the decreased frequency of the push resonator was 131.99 Hz. The total frequency shift was therefore translated to a sensitivity of 258.77 Hz/g, only 3.6% higher than the calculation of 249.46 Hz/g.

**Figure 9:**
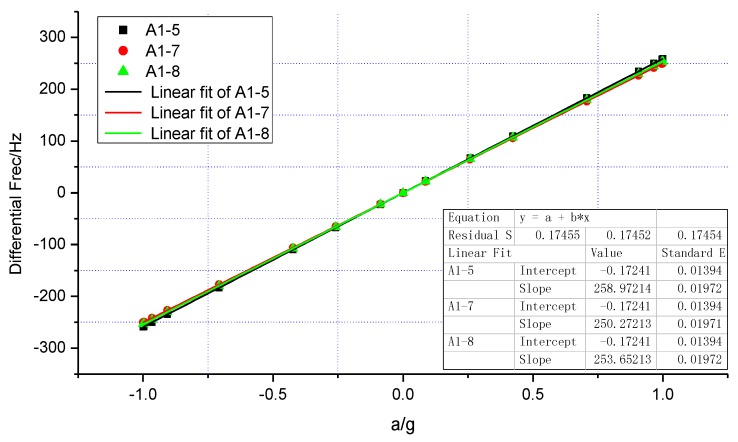
Variation of the differential resonant frequency Δf for three SMRA prototypes between the resonators in the range of ±1 g.

**Figure 10:**
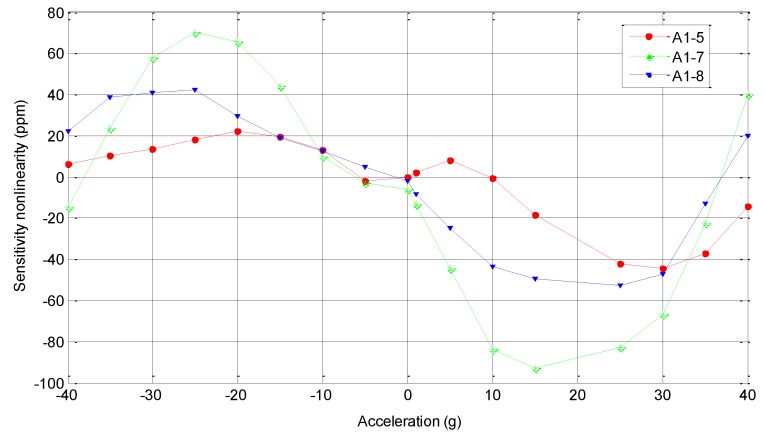
Testing nonlinearity of the sensitivity in the range of ±40 g.

Figure 9 shows experimental points and a linear fitting of the measured differential frequency for the acceleration of sin(θ) g on the three SMRA prototypes: A1-5, A1-7, A1-8. The rotating angle θ was adjusted to be 0°, 5°, 15°, 25°, 45°, 65°, 75°, 85°, 90°, 95°, 105°, 115°, 135°, 155°, 165°, 175°, 180°, 185°, 195°, 205°, 225°, 245°, 255°, 265°, 270°, 275°, 285°, 295°, 315°, 335°, 345°, and 355°, respectively [23]. Good linearity of these prototypes is observed in this range of operation. By fitting the 32 sets of the differential frequency, the average sensitivity within 1 g turns out to be 254.3 Hz/g. As shown in Figure 10, when the SMRA prototypes were subjected to an input acceleration in the range of ±40 g with a constant temperature control, the testing nonlinearity of the sensitivity is within 100 ppm. All of the above results have helped to confirm our theory.

**Figure 11:**
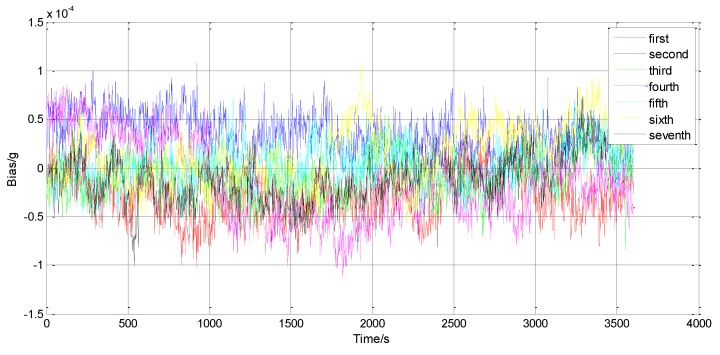
Measured bias (magnified seven times) *versus* the elapsed time.

**Figure 12:**
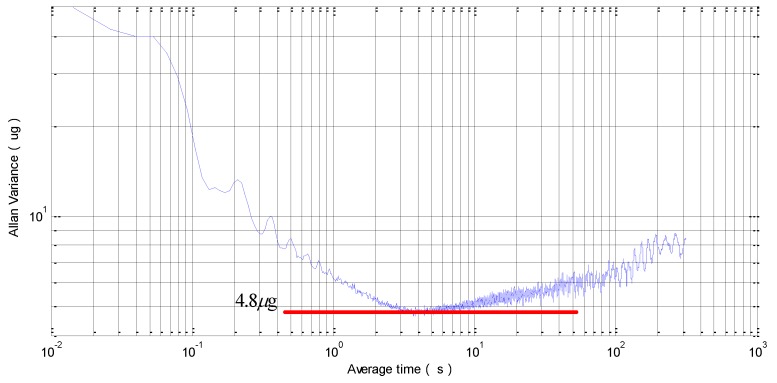
Measured Allan variance.

To study the bias stability, the A1-5’s input axis was kept horizontal to insure that the input accelerometer was 0 g, and then the whole accelerometer was kept powered for 20 min. In this working state, the output data of this prototype was recorded at a 1 Hz sampling rate for 60 min. To avoid a temperature influence, the sample had been put on a rotating platform under a constant 20 °C. Then, the above steps were repeated for seven times. All the tested data have been presented in Figure 11 with a one-hour bias stability of 55 μg and a bias repeatability of 48 μg. The random bias variance was then characterized using Allan variance, a method proposed for clock systems [24]. Allan variance calculation is applied to the frequency reading and plotted in Figure 12. The Allan variance flattens around 3 s and then shows an increase trend as the averaging time increases. The flatten floor is known as the Allan deviation, which indicates the random parts of the bias-instability is 4.8 μg. The increase trend part is believed to be caused by the temperature drift.

As a result, compared to the studies of [2,11,25,26], after reasonable geometrical design, the SMRA reported in this paper stands out for its high-sensitivity of over 210 Hz/g, the input range of ±40 g, one-hour bias stability of 55 μg and the bias repeatability of 48 μg.

## 5. Conclusions/Outlook

This paper presents the design and experimental evaluation of an SMRA. We apply energy-consumed concept and the Nelder-Mead algorithm on this sensor to address the design issues and to increase its sensitivity. This SOI-MEMS fabricated SMRA has a closed-form sensitivity of 211.5 Hz/g, its FEM value is 203 Hz/g, and the experimental value is 254.3 Hz/g. The nonlinearity of the *S*_g_ is below 100 ppm within the input range of ±40 g. All the results exhibit good agreement with the theoretical results. The sensitivity of the SMRA has increased 66% compared to the previous work by using a novel optimization algorithm. This improvement is mainly attributed to both the re-designed DETF and the reduced energy loss on the micro-lever. All the above work provides a reference for the geometrical design of other MEMS sensors.

Other key performances like bias stability, bias repeatability, and Allan variance are also shown in the paper. It should be noted that the testing results are prone to temperature shifts. Therefore, how temperature and residual stress influence the SMRA’s performance remain to be elucidated, and this will be explored in the future work. A careful study on the model for the thermal stress of SMRAs is now under way.

## Appendix A

By supposing an inertial load *a* is applied to the SMRA, the ends of the input and output beams are loaded with vertical forces *F_yi_* (for the input beam) and *F_yo_* (for the output beam), horizontal forces *F*_xi_ (for the input beam) and *F*_xo_ (for the output beam) and bending moments *M_i_* (for the input beam) and *M_o_* (for the output beam). The axial force and moment of each beam on the micro-lever in Figure 5b are shown in Table A1.

**Table A1:** The axial force and moment of each beam on the micro-lever.

Beam Number	Axial Force *F_j_*	Moment *M_j_* (*j* = 1, 2, 3, 4, 5)
1	$F_{1}=-F_{yi}$	$M_{1}\left(x\right)=M_{i}+F_{xi}x$
2	$F_{2}=F_{xi}$	$M_{2}\left(x\right)=M_{i}+F_{xi}l_{i}+F_{yi}x$
3	$F_{3}=F_{yo}$	$M_{3}\left(x\right)=M_{o}-F_{xo}x$
4	$F_{4}=F_{xi}+F_{xo}$	$M_{4}\left(x\right)=M_{i}+F_{xi}l_{i}+F_{yi}\left(x+l_{in}-l_{out}\right)+M_{o}-F_{xo}l_{o}+F_{yo}x$
5	$F_{5}=-\left(F_{yi}+F_{yo}\right)$	$M_{5}\left(x\right)=M_{i}+F_{xi}\left(l_{i}+x\right)+F_{yi}\left(x+l_{in}\right)+M_{o}-F_{xo}\left(l_{o}-x\right)+F_{yo}l_{out}$

According to the theory of Castigliano’s method [27], the displacements and rotation angles of the input and output beams can be expressed by the following equations:

(A1) $$\begin{array}{ll} d_{xi} & =\int_{0}^{l_{i}}\left(\frac{M_{1}}{EI_{i}}\cdot \frac{\partial M_{1}}{\partial F_{xi}}+\frac{F_{1}}{Eq_{i}}\cdot \frac{\partial F_{1}}{\partial F_{xi}}\right)dx+\int_{0}^{l_{in}-l_{out}}\left(\frac{M_{2}}{EI_{lever}}\cdot \frac{\partial M_{2}}{\partial F_{xi}}+\frac{F_{2}}{Eq_{lever}}\cdot \frac{\partial F_{2}}{\partial F_{xi}}\right)dx+\int_{0}^{l_{o}}\left(\frac{M_{3}}{EI_{o}}\cdot \frac{\partial M_{3}}{\partial F_{xi}}+\frac{F_{3}}{Eq_{o}}\cdot \frac{\partial F_{3}}{\partial F_{xi}}\right)dx\\ & +\int_{0}^{l_{out}}\left(\frac{M_{4}}{EI_{lever}}\cdot \frac{\partial M_{4}}{\partial F_{xi}}+\frac{F_{4}}{Eq_{lever}}\cdot \frac{\partial F_{4}}{\partial F_{xi}}\right)dx+\int_{0}^{l_{p}}\left(\frac{M_{5}}{EI_{p}}\cdot \frac{\partial M_{5}}{\partial F_{xi}}+\frac{F_{5}}{Eq_{p}}\cdot \frac{\partial F_{5}}{\partial F_{xi}}\right)dx\\ = & \frac{1}{6E}\left(\begin{array}{l} \frac{2F_{xi}l_{i}^{3}+3l_{i}^{2}M_{i}}{I_{i}}+\frac{-3l_{i}{\left(F_{xi}l_{i}+M_{i}\right)}^{2}+3l_{i}{\left(F_{yi}l_{in}+F_{xi}l_{i}+M_{i}\right)}^{3}}{F_{yi}I_{lever}}\\ +\frac{-3l_{i}{\left(F_{yi}l_{in}+F_{xi}l_{i}-F_{xo}l_{o}+M_{i}+M_{o}\right)}^{2}+3l_{i}{\left(F_{yo}l_{out}+F_{yi}\left(l_{in}+l_{out}\right)+F_{xi}l_{i}-F_{xo}l_{o}+M_{i}+M_{o}\right)}^{2}}{\left(F_{yi}+F_{yo}\right)I_{lever}}\\ +\frac{-3l_{i}{\left(F_{yo}l_{out}+F_{yi}\left(l_{in}+l_{out}\right)+F_{xi}l_{i}-F_{xo}l_{o}+M_{i}+M_{o}\right)}^{2}+3\left(l_{i}+l_{p}\right){\left(F_{yo}l_{out}+F_{yi}\left(l_{in}+l_{out}\right)+F_{xi}l_{i}-F_{xo}l_{o}+\left(F_{xi}+F_{xo}\right)l_{p}+M_{i}+M_{o}\right)}^{2}}{\left(F_{xi}+F_{xo}\right)I_{p}}\\ +\frac{-{\left(F_{yo}l_{out}+F_{yi}\left(l_{in}+l_{out}\right)+F_{xi}l_{i}-F_{xo}l_{o}+M_{i}+M_{o}\right)}^{3}+{\left(F_{yo}l_{out}+F_{yi}\left(l_{in}+l_{out}\right)+F_{xi}l_{i}-F_{xo}l_{o}+\left(F_{xi}+F_{xo}\right)l_{p}+M_{i}+M_{o}\right)}^{3}}{{\left(F_{xi}+F_{xo}\right)}^{2}I_{p}} \end{array}\right) \end{array}$$

(A2) $$\begin{array}{ll} d_{yi} & =\int_{0}^{l_{i}}\left(\frac{M_{1}}{EI_{i}}\cdot \frac{\partial M_{1}}{\partial F_{yi}}+\frac{F_{1}}{Eq_{i}}\cdot \frac{\partial F_{1}}{\partial F_{yi}}\right)dx+\int_{0}^{l_{in}-l_{out}}\left(\frac{M_{2}}{EI_{lever}}\cdot \frac{\partial M_{2}}{\partial F_{yi}}+\frac{F_{2}}{Eq_{lever}}\cdot \frac{\partial F_{2}}{\partial F_{yi}}\right)dx+\int_{0}^{l_{o}}\left(\frac{M_{3}}{EI_{o}}\cdot \frac{\partial M_{3}}{\partial F_{yi}}+\frac{F_{3}}{Eq_{o}}\cdot \frac{\partial F_{3}}{\partial F_{yi}}\right)dx\\ & +\int_{0}^{l_{out}}\left(\frac{M_{4}}{EI_{lever}}\cdot \frac{\partial M_{4}}{\partial F_{yi}}+\frac{F_{4}}{Eq_{lever}}\cdot \frac{\partial F_{4}}{\partial F_{yi}}\right)dx+\int_{0}^{l_{p}}\left(\frac{M_{5}}{EI_{p}}\cdot \frac{\partial M_{5}}{\partial F_{yi}}+\frac{F_{5}}{Eq_{p}}\cdot \frac{\partial F_{5}}{\partial F_{yi}}\right)dx\\ = & \frac{1}{6E}\left(\begin{array}{l} \frac{3l_{in}{\left(F_{yi}l_{in}+F_{xi}l_{i}+M_{i}\right)}^{2}}{F_{yi}I_{lever}}+\frac{{\left(F_{xi}l_{i}+M_{i}\right)}^{2}-{\left(F_{yi}l_{in}+F_{xi}l_{i}+M_{i}\right)}^{3}}{F_{yi}^{2}I_{lever}}-\frac{3l_{in}{\left(F_{yi}l_{in}+F_{xi}l_{i}-F_{xo}l_{o}+M_{i}+M_{o}\right)}^{2}}{\left(F_{yi}+F_{yo}\right)I_{lever}}\\ +\frac{3\left(l_{in}+l_{out}\right){\left(F_{yo}l_{out}+F_{yi}\left(l_{in}+l_{out}\right)+F_{xi}l_{i}-F_{xo}l_{o}+M_{i}+M_{o}\right)}^{2}}{\left(F_{yi}+F_{yo}\right)I_{lever}}-\frac{{\left(F_{yi}l_{in}+F_{xi}l_{i}-F_{xo}l_{o}+M_{i}+M_{o}\right)}^{3}}{{\left(F_{yi}+F_{yo}\right)}^{2}I_{lever}}\\ +\frac{{\left(F_{yo}l_{out}+F_{yi}\left(l_{in}+l_{out}\right)+F_{xi}l_{i}-F_{xo}l_{o}+M_{i}+M_{o}\right)}^{3}}{{\left(F_{yi}+F_{yo}\right)}^{2}I_{lever}}-\frac{3\left(l_{in}+l_{out}\right){\left(F_{yo}l_{out}+F_{yi}\left(l_{in}+l_{out}\right)+F_{xi}l_{i}-F_{xo}l_{o}+M_{i}+M_{o}\right)}^{2}}{\left(F_{xi}+F_{xo}\right)I_{p}}\\ +\frac{3\left(l_{in}+l_{out}\right){\left(F_{yo}l_{out}+F_{yi}\left(l_{in}+l_{out}\right)+F_{xi}l_{i}-F_{xo}l_{o}+\left(F_{xi}+F_{xo}\right)l_{p}+M_{i}+M_{o}\right)}^{2}}{\left(F_{xi}+F_{xo}\right)I_{p}} \end{array}\right) \end{array}$$

(A3) $$\begin{array}{ll} \theta_{i} & =\int_{0}^{l_{i}}\left(\frac{M_{1}}{EI_{i}}\cdot \frac{\partial M_{1}}{\partial M_{i}}+\frac{F_{1}}{Eq_{i}}\cdot \frac{\partial F_{1}}{\partial M_{i}}\right)dx+\int_{0}^{l_{in}-l_{out}}\left(\frac{M_{2}}{EI_{lever}}\cdot \frac{\partial M_{2}}{\partial M_{i}}+\frac{F_{2}}{Eq_{lever}}\cdot \frac{\partial F_{2}}{\partial M_{i}}\right)dx+\int_{0}^{l_{o}}\left(\frac{M_{3}}{EI_{o}}\cdot \frac{\partial M_{3}}{\partial M_{i}}+\frac{F_{3}}{Eq_{o}}\cdot \frac{\partial F_{3}}{\partial M_{i}}\right)dx\\ & +\int_{0}^{l_{out}}\left(\frac{M_{4}}{EI_{lever}}\cdot \frac{\partial M_{4}}{\partial M_{i}}+\frac{F_{4}}{Eq_{lever}}\cdot \frac{\partial F_{4}}{\partial M_{i}}\right)dx+\int_{0}^{l_{p}}\left(\frac{M_{5}}{EI_{p}}\cdot \frac{\partial M_{5}}{\partial M_{i}}+\frac{F_{5}}{Eq_{p}}\cdot \frac{\partial F_{5}}{\partial M_{i}}\right)dx\\ = & \frac{1}{6E}\left(\begin{array}{l} \frac{3F_{xi}l_{i}^{2}+6l_{i}M_{i}}{I_{i}}+\frac{-3{\left(F_{xi}l_{i}+M_{i}\right)}^{2}+3{\left(F_{yi}l_{in}+F_{xi}l_{i}+M_{i}\right)}^{2}}{F_{yi}I_{lever}}-\frac{3{\left(F_{yi}l_{in}+F_{xi}l_{i}-F_{xo}l_{o}+M_{i}+M_{o}\right)}^{2}}{\left(F_{yi}+F_{yo}\right)I_{lever}}\\ +\frac{3{\left(F_{yo}l_{out}+F_{yi}\left(l_{in}+l_{out}\right)+F_{xi}l_{i}-F_{xo}l_{o}+\left(F_{xi}+F_{xo}\right)l_{p}+M_{i}+M_{o}\right)}^{2}}{\left(F_{yi}+F_{yo}\right)I_{lever}}-\frac{3{\left(F_{yo}l_{out}+F_{yi}\left(l_{in}+l_{out}\right)+F_{xi}l_{i}-F_{xo}l_{o}+M_{i}+M_{o}\right)}^{2}}{\left(F_{xi}+F_{xo}\right)I_{p}}\\ +\frac{3{\left(F_{yo}l_{out}+F_{yi}\left(l_{in}+l_{out}\right)+F_{xi}l_{i}-F_{xo}l_{o}+\left(F_{xi}+F_{xo}\right)l_{p}+M_{i}+M_{o}\right)}^{2}}{\left(F_{xi}+F_{xo}\right)I_{p}} \end{array}\right) \end{array}$$

(A4) $$\begin{array}{ll} d_{xo} & =\int_{0}^{l_{i}}\left(\frac{M_{1}}{EI_{i}}\cdot \frac{\partial M_{1}}{\partial F_{xo}}+\frac{F_{1}}{Eq_{i}}\cdot \frac{\partial F_{1}}{\partial F_{xo}}\right)dx+\int_{0}^{l_{in}-l_{out}}\left(\frac{M_{2}}{EI_{lever}}\cdot \frac{\partial M_{2}}{\partial F_{xo}}+\frac{F_{2}}{Eq_{lever}}\cdot \frac{\partial F_{2}}{\partial F_{xo}}\right)dx+\int_{0}^{l_{o}}\left(\frac{M_{3}}{EI_{o}}\cdot \frac{\partial M_{3}}{\partial F_{xo}}+\frac{F_{3}}{Eq_{o}}\cdot \frac{\partial F_{3}}{\partial F_{xo}}\right)dx\\ & +\int_{0}^{l_{out}}\left(\frac{M_{4}}{EI_{lever}}\cdot \frac{\partial M_{4}}{\partial F_{xo}}+\frac{F_{4}}{Eq_{lever}}\cdot \frac{\partial F_{4}}{\partial F_{xo}}\right)dx+\int_{0}^{l_{p}}\left(\frac{M_{5}}{EI_{p}}\cdot \frac{\partial M_{5}}{\partial F_{xo}}+\frac{F_{5}}{Eq_{p}}\cdot \frac{\partial F_{5}}{\partial F_{xo}}\right)dx\\ = & \frac{1}{6E}\left(\begin{array}{l} \frac{l_{o}^{2}\left(2F_{xo}l_{o}-3M_{o}\right)}{I_{o}}+\frac{3l_{o}{\left(F_{yi}l_{in}+F_{xi}l_{i}-F_{xo}l_{o}+M_{i}+M_{o}\right)}^{2}-3l_{o}{\left(F_{yo}l_{out}+F_{yi}\left(l_{in}+l_{out}\right)+F_{xi}l_{i}-F_{xo}l_{o}+M_{i}+M_{o}\right)}^{2}}{\left(F_{yi}+F_{yo}\right)I_{lever}}\\ +\frac{3l_{o}{\left(F_{yo}l_{out}+F_{yi}\left(l_{in}+l_{out}\right)+F_{xi}l_{i}-F_{xo}l_{o}+M_{i}+M_{o}\right)}^{2}+3\left(l_{p}-l_{o}\right){\left(F_{yo}l_{out}+F_{yi}\left(l_{in}+l_{out}\right)+F_{xi}l_{i}-F_{xo}l_{o}+\left(F_{xi}+F_{xo}\right)l_{p}+M_{i}+M_{o}\right)}^{2}}{\left(F_{xi}+F_{xo}\right)I_{p}}\\ +\frac{-{\left(F_{yo}l_{out}+F_{yi}\left(l_{in}+l_{out}\right)+F_{xi}l_{i}-F_{xo}l_{o}+M_{i}+M_{o}\right)}^{3}+{\left(F_{yo}l_{out}+F_{yi}\left(l_{in}+l_{out}\right)+F_{xi}l_{i}-F_{xo}l_{o}+\left(F_{xi}+F_{xo}\right)l_{p}+M_{i}+M_{o}\right)}^{3}}{{\left(F_{xi}+F_{xo}\right)}^{2}I_{p}} \end{array}\right) \end{array}$$

(A5) $$\begin{array}{ll} d_{yo} & =\int_{0}^{l_{i}}\left(\frac{M_{1}}{EI_{i}}\cdot \frac{\partial M_{1}}{\partial F_{yo}}+\frac{F_{1}}{Eq_{i}}\cdot \frac{\partial F_{1}}{\partial F_{yo}}\right)dx+\int_{0}^{l_{in}-l_{out}}\left(\frac{M_{2}}{EI_{lever}}\cdot \frac{\partial M_{2}}{\partial F_{yo}}+\frac{F_{2}}{Eq_{lever}}\cdot \frac{\partial F_{2}}{\partial F_{yo}}\right)dx+\int_{0}^{l_{o}}\left(\frac{M_{3}}{EI_{o}}\cdot \frac{\partial M_{3}}{\partial F_{yo}}+\frac{F_{3}}{Eq_{o}}\cdot \frac{\partial F_{3}}{\partial F_{yo}}\right)dx\\ & +\int_{0}^{l_{out}}\left(\frac{M_{4}}{EI_{lever}}\cdot \frac{\partial M_{4}}{\partial F_{yo}}+\frac{F_{4}}{Eq_{lever}}\cdot \frac{\partial F_{4}}{\partial F_{yo}}\right)dx+\int_{0}^{l_{p}}\left(\frac{M_{5}}{EI_{p}}\cdot \frac{\partial M_{5}}{\partial F_{yo}}+\frac{F_{5}}{Eq_{p}}\cdot \frac{\partial F_{5}}{\partial F_{yo}}\right)dx\\ = & \frac{1}{6E}\left(\begin{array}{l} \frac{3l_{out}{\left(F_{yo}l_{out}+F_{yi}\left(l_{in}+l_{out}\right)+F_{xi}l_{i}-F_{xo}l_{o}+M_{i}+M_{o}\right)}^{2}}{\left(F_{yi}+F_{yo}\right)I_{lever}}+\frac{{\left(F_{yi}l_{in}+F_{xi}l_{i}-F_{xo}l_{o}+M_{i}+M_{o}\right)}^{3}}{{\left(F_{yi}+F_{yo}\right)}^{2}I_{p}}\\ -\frac{{\left(F_{yo}l_{out}+F_{yi}\left(l_{in}+l_{out}\right)+F_{xi}l_{i}-F_{xo}l_{o}+M_{i}+M_{o}\right)}^{3}}{{\left(F_{yi}+F_{yo}\right)}^{2}I_{p}}-\frac{3l_{out}{\left(F_{yo}l_{out}+F_{yi}\left(l_{in}+l_{out}\right)+F_{xi}l_{i}-F_{xo}l_{o}+M_{i}+M_{o}\right)}^{2}}{\left(F_{xi}+F_{xo}\right)I_{p}}\\ +\frac{3l_{out}{\left(F_{yo}l_{out}+F_{yi}\left(l_{in}+l_{out}\right)+F_{xi}l_{i}-F_{xo}l_{o}+\left(F_{xi}+F_{xo}\right)l_{p}+M_{i}+M_{o}\right)}^{2}}{\left(F_{xi}+F_{xo}\right)I_{p}} \end{array}\right) \end{array}$$

(A6) $$\begin{array}{ll} \theta_{o} & =\int_{0}^{l_{i}}\left(\frac{M_{1}}{EI_{i}}\cdot \frac{\partial M_{1}}{\partial M_{o}}+\frac{F_{1}}{Eq_{i}}\cdot \frac{\partial F_{1}}{\partial M_{o}}\right)dx+\int_{0}^{l_{in}-l_{out}}\left(\frac{M_{2}}{EI_{lever}}\cdot \frac{\partial M_{2}}{\partial M_{o}}+\frac{F_{2}}{Eq_{lever}}\cdot \frac{\partial F_{2}}{\partial M_{o}}\right)dx+\int_{0}^{l_{o}}\left(\frac{M_{3}}{EI_{o}}\cdot \frac{\partial M_{3}}{\partial M_{o}}+\frac{F_{3}}{Eq_{o}}\cdot \frac{\partial F_{3}}{\partial M_{o}}\right)dx\\ & +\int_{0}^{l_{out}}\left(\frac{M_{4}}{EI_{lever}}\cdot \frac{\partial M_{4}}{\partial M_{o}}+\frac{F_{4}}{Eq_{lever}}\cdot \frac{\partial F_{4}}{\partial M_{o}}\right)dx+\int_{0}^{l_{p}}\left(\frac{M_{5}}{EI_{p}}\cdot \frac{\partial M_{5}}{\partial M_{o}}+\frac{F_{5}}{Eq_{p}}\cdot \frac{\partial F_{5}}{\partial M_{o}}\right)dx\\ = & \frac{1}{6E}\left(\begin{array}{l} \frac{-3F_{xo}l_{o}^{2}+6l_{o}M_{o}}{I_{o}}-\frac{3{\left(F_{yi}l_{in}+F_{xi}l_{i}-F_{xo}l_{o}+M_{i}+M_{o}\right)}^{2}}{\left(F_{yi}+F_{yo}\right)I_{lever}}\\ +\frac{3{\left(F_{yo}l_{out}+F_{yi}\left(l_{in}+l_{out}\right)+F_{xi}l_{i}-F_{xo}l_{o}+M_{i}+M_{o}\right)}^{2}}{\left(F_{yi}+F_{yo}\right)I_{lever}}-\frac{3{\left(F_{yo}l_{out}+F_{yi}\left(l_{in}+l_{out}\right)+F_{xi}l_{i}-F_{xo}l_{o}+M_{i}+M_{o}\right)}^{2}}{\left(F_{xi}+F_{xo}\right)I_{p}}\\ +\frac{3{\left(F_{yo}l_{out}+F_{yi}\left(l_{in}+l_{out}\right)+F_{xi}l_{i}-F_{xo}l_{o}+\left(F_{xi}+F_{xo}\right)l_{p}+M_{i}+M_{o}\right)}^{2}}{\left(F_{xi}+F_{xo}\right)I_{p}} \end{array}\right) \end{array}$$

where *I* is the beam’s bending moment in the x-y plane, *l* is the beam length and *q* is the beam cross-section with a subscript *i*, *o*, *p*, and *lever* representing the input beam, the output beam, the pivot beam, and the lever arm; *l*_out_ and *l_in_* are the input and output arm length of the micro-lever.

Applying force to the proof mass leads to the following equation:

(A7) $$\frac{m_{1}a}{4}=F_{yi}+K_{1}d_{yi}$$

Moreover, the proof mass can be regarded as rigid, and therefore, the boundary conditions at the end of input beam can be expressed as

(A8) $$d_{xi}=d_{zi}=0$$

(A9) $$d_{yi}=\left(m_{1}a-4F_{yi}\right)/4K_{1}$$

Similarly, the boundary conditions at the end of output beam can be expressed as

(A10) $$d_{xo}=d_{zo}=0$$

(A11) $$d_{yo}=-F_{yo}/K_{2}$$

where *K*_2_ is equal to the spring constant of one DETF beam *k_f_* connected in series with the spring constant of the half connecting mass *k_b_*.

By solving these boundary conditions for *F*_xi_, *F_yi_*, *M_i_*, *F_yo_*, *F*_xo_ and *M_o_*, the effective amplification factor can be obtained as

(A12) $$A^{*}=\frac{F_{yo}}{m_{1}a/4}$$

Using the energy method to calculate the spring constant *K*_1_ along the input axis of one flexure [28] (see Figure 1), this constant can be obtained to be

(A13) $$K_{1}=\frac{3EI_{a}I_{b}(3I_{a}I_{b}+2I_{a}(l_{b1}+l_{b2})}{\left(6I_{b}^{2}l_{a}^{2}l_{b2}^{2}+6I_{a}I_{b}l_{a}(l_{b1}^{3}-l_{b1}^{2}l_{b2}+l_{b2}^{2}l_{b1}+l_{b2}^{3})+I_{a}^{2}(l_{b1}^{4}+4l_{b1}^{3}l_{b2}-6l_{b1}^{2}l_{b2}^{2}+4l_{b1}l_{b2}^{3}+l_{b2}^{4}\right)}$$

where *I* is inertia moment around the z-axis of each flexure beam with *a* and *b* as the beams corresponding to *l*_a_, *l*_*b*1_ and *l*_*b*2_.

According to beam bending theory,

(A14) $$k_{f}=\frac{Ewt}{l}$$

The connecting mass has a high width-length ratio (more than 1:5), and it can be defined as a short beam. The deformation of the half connecting mass is shown in Figure 5. Its boundary conditions can be simplified to those of a simply supported beam. By using *Timoshenko* beam theory [29], the maximum deflection of the half connecting mass can be described to be

(A15) $$\omega_{M}=\frac{F_{yo}L_{c}^{3}}{24EI_{c}}(1+\frac{12a_{s}EI_{c}}{Gw_{c}tL_{c}^{2}})$$

where *E* is the Young’s modulus; *G* is the shear modulus; *L_c_*, *w_c_*, *t* and *I_c_* is the length, width, thickness and cross-sectional inertia moment across the z-axis of the half connecting mass, respectively; and *a_s_* is the shear coefficient of rectangular cross-section.

By substituting single-crystal silicon material parameters into Equation (A15),

(A16) $$\omega_{M}=\frac{F_{yo}}{2Et}{\left(\frac{L_{c}}{w_{c}}\right)}^{3}\left(1+3.81{\left(\frac{w_{c}}{L_{c}}\right)}^{2}\right)$$

Then, the spring constant of the half connecting mass along the input axis can be expressed as

(A17) $$k_{c}=\frac{F_{yo}}{\omega_{M}}=\frac{2Et{\left(w_{c}/L_{c}\right)}^{3}}{1+3.81{\left(w_{c}/L_{c}\right)}^{2}}$$

and the spring constant of *K*_2_ is

(A18) $$K_{2}=1/\left(\frac{1}{k_{f}}+\frac{1}{k_{c}}\right)=\frac{2Etw_{c}^{3}w_{f}}{2l_{f}w_{c}^{3}+L_{c}^{3}w_{f}+3.81L_{c}w_{c}^{2}w_{f}}$$

The calculated value of *K*_c_ and *K*_f_ are 5.84 × 10^5^ N/m and 0.98 × 10^5^ N/m.

## References

[1] Marek J., Gómez U.M. (2012). MEMS (micro-electro-mechanical systems) for automotive and consumer electronics. Chips A Guide to the Future of Nanoelectronics the Frontiers Collection.

[2] Seshia A.A., Palaniapan M., Roessig T.A., Howe R.T., Gooch R.W., Schimert T.R., Montague S. (2002). A vacuum packaged surface micromachined resonant accelerometer. J. Microelectromech. Syst..

[3] Comi C., Corigliano A., Langfelder G., Longoni A., Tocchio A., Simoni B. (2010). A resonant microaccelerometer with high sensitivity operating in an oscillating circuit. J. Microelectromech. Syst..

[4] Pinto D., Mercier D., Kharrat C., Colinet E., Nguyen V., Reig B., Hentz S. (2009). A small and high sensitivity resonant accelerometer. Procedia Chem..

[5] Shi R., Jia F.-X., Qiu A.-P., Su Y. (2013). Phase noise analysis of micromechanical silicon resonant accelerometer. Sens. Actuators A Phys..

[6] Chae J., Kulah H., Najafi K. (2005). A cmos-compatible high aspect ratio silicon-on-glass in-plane micro-accelerometer. J. Micromech. Microeng..

[7] Fan K., Che L., Xiong B., Wang Y. (2007). A silicon micromachined high-shock accelerometer with a bonded hinge structure. J. Micromech. Microeng..

[8] Krishnamoorthy U., Olsson R., Bogart G.R., Baker M., Carr D., Swiler T., Clews P. (2008). In-plane mems-based nano-g accelerometer with sub-wavelength optical resonant sensor. Sens. Actuators A Phys..

[9] Zou X., Thiruvenkatanathan P., Seshia A.A. (2014). A seismic-grade resonant mems accelerometer. J. Microelectromech. Syst..

[10] Zou X., Thiruvenkatanathan P., Seshia A.A. Micro-electro-mechanical resonant tilt sensor. Proceedings of the 2012 IEEE International Frequency Control Symposium (FCS).

[11] Su S.X., Yang H.S., Agogino A.M. (2005). A resonant accelerometer with two-stage microleverage mechanisms fabricated by soi-mems technology. IEEE Sens. J..

[12] Xia G.-M., Qiu A.-P., Shi Q., Su Y. Test and evaluation of a silicon resonant accelerometer implemented in soi technology. Proceedings of the 2013 IEEE Sensors.

[13] Dong J.-H., Qiu A.-P., Shi R. Temperature influence mechanism of micromechanical silicon oscillating accelerometer. Proceedings of the 2011 IEEE Power Engineering And Automation Conference (PEAM).

[14] Shi R., Jiang S., Qiu A.-P., Su Y. (2011). Application of microlever to micromechanical silicon resonant accelerometers. Opt. Precis. Eng..

[15] Lagarias J.C., Reeds J.A., Wright M.H., Wright P.E. (1998). Convergence properties of the Nelder-Mead simplex method in low dimensions. SIAM J. Optim..

[16] Nelder J.A., Mead R. (1965). A simplex method for function minimization. Comput. J..

[17] Brosnihan T.J., Bustillo J.M., Pisano A.P., Howe R.T. Embedded interconnect and electrical isolation for high-aspect-ratio, soi inertial instruments. Proceedings of the 1977 Internatonal Conference Solid State Sensors and Actuators, Transducers ’97.

[18] Torunbalci M.M., Alper S.E., Akin T. Wafer level hermetic encapsulation of mems inertial sensors using soi cap wafers with vertical feedthroughs. Proceedings of the 2014 International Symposium on Inertial Sensors and Systems (ISISS).

[19] Renard S. (2000). SOI micromachining technologies for MEMS. Micromachining and Microfabrication.

[20] Lin C.-W., Hsu C.-P., Yang H.-A., Wang W.C., Fang W. (2008). Implementation of silicon-on-glass mems devices with embedded through-wafer silicon vias using the glass reflow process for wafer-level packaging and 3D chip integration. J. Micromech. Microeng..

[21] Harris C.M., Piersol A.G., Paez T.L. (2002). Harris’ Shock and Vibration Handbook.

[22] Roessig T.-A.W. (1998). Integrated Mems Tuning Fork Oscillators for Sensor Applications.

[23] IEEE (1999). 1293–1998—IEEE standard specification format guide and test procedure for linear, single-axis, non-gyroscopic accelerometers.

[24] Allan D.W. (1987). Time and frequency (time-domain) characterization, estimation, and prediction of precision clocks and oscillators. IEEE Trans. Ultrason. Ferroelectr. Freq. Control.

[25] Lefort O., Jaud S., Quer R., Milesi A. Inertial grade silicon vibrating beam accelerometer. Proceedings of Inertial Sensors and Systems 2012.

[26] He L., Xu Y.P., Palaniapan M. (2008). A cmos readout circuit for soi resonant accelerometer with 4-bias stability and 20-resolution. Solid-State Circuits, IEEE J..

[27] Argyris J.H., Kelsey S. (1960). Energy Theorems and Structural Analysis.

[28] Iyer S.V. (2003). Modeling and Simulation of Non-Idealities in a Z-Axis Cmos-Mems Gyroscope. Ph.D. Thesis.

[29] Timoshenko S., Woinowsky-Krieger S., Woinowsky-Krieger S. (1959). Theory of Plates and Shells.

